# The Contribution of Mutual Grooming to Affiliative Relationships in a Feral Misaki Horse Herd

**DOI:** 10.3390/ani10091564

**Published:** 2020-09-03

**Authors:** Masaki Shimada, Nae Suzuki

**Affiliations:** Department of Animal Sciences, Teikyo University of Science, 2525 Yatsusawa, Yamanashi prefecture, Uenohara 409-0193, Japan; agu_horse0731@yahoo.co.jp

**Keywords:** *Equus caballus*, Misaki feral horse, affiliative relationship, mutual grooming, social rank

## Abstract

**Simple Summary:**

Social grooming strengthens affiliative relationships between participants in many social primates. Three hypotheses regarding the function of mutual grooming in feral horses were tested: the affiliative relationship strengthening hypothesis, the worsened relationship restoring hypothesis, and the parasite removal hypothesis. All the nine horses in the “6m” herd in a Misaki feral horse (*Equus caballus*) herd in Cape Toi, Japan, were investigated in terms of kinship, grooming, aggression, proximity, social rank, and social network. The correlations between mutual grooming and proximity and between aggression and proximity were established mathematically. Controlling for kinship, there were significant positive partial correlations between mutual grooming and proximity and between aggression and proximity. No correlation was observed between aggression and mutual grooming. Individuals that spent less time on self-grooming invested longer times receiving grooming from other individuals. In a feral horse population, mutual grooming maintains hygiene by controlling ectoparasites and forges affiliative interactions between herd members.

**Abstract:**

Although herd size, structure, stability, and social rank among Misaki feral horses have been reported, no studies have been conducted on the affiliative relationships and interactions among members in a Misaki horse herd. The validity of three hypotheses regarding the function of social grooming, the affiliative relationship strengthening hypothesis, the worsened relationship restoring hypothesis, and the grooming parasite removal hypothesis, were tested in a Misaki feral horse (*Equus caballus*) herd in Cape Toi, Japan. All the nine horses in the “6m” herd were investigated in terms of kinship, grooming, aggression, proximity, social rank, and social network. Mutual grooming occurred only in pairs and was almost perfectly symmetrical. For each member, there was a significant negative correlation between total grooming received from other individuals and self-grooming. Controlling for kinship, there were significant positive partial correlations between mutual grooming and proximity and between aggression and proximity. No correlation was observed between aggression and mutual grooming. The results suggest that mutual grooming symmetry may contribute that both participants simultaneously benefit from parasite removal and strengthen affiliative relationships between seasonally changing herd members; however, mutual grooming did not foster restoring the worsened relationship following aggression promoted by physical proximity. The findings of this study may elucidate the mechanisms by which interactions between herd members are maintained or strengthened.

## 1. Introduction

In many mammals, individuals strengthen affiliative relationships with unrelated individuals to enhance their reproductive success [1,2,3]. Social grooming strengthens affiliative relationships between participants in many social primates [4,5,6]. Social grooming between primates is primarily asymmetrical [7,8]. Grooming exchanges continue over extended periods in groups with long-term stability [9,10,11]. Pairs that equalize the amount of social grooming in the long term maintain their affiliative relationships [5,6].

Horses (*Equus caballus*) that have left human control and live in environments with minimal artificial control are known as feral [12]. Feral horses live in herds comprising one to a few adult males (stallions), several unrelated adult females (mares), and immature offspring of both sexes (fillies and colts) [13,14,15,16,17]. The social rank established among herd members shows long-term stability [18,19,20]. Both colts and fillies leave their natal herds at approximately 2–3 years of age. Fillies either remain unaffiliated with any one particular herd or migrate to another [21,22]. Colts either form bachelor herds [23,24,25,26] or will sequester mares to form their own herd [22]. Herd membership is stable during the breeding season between spring and autumn [25,27]; however, as in the case of Misaki feral horses in Japan, a herd may disperse in the winter, and its membership might fluctuate by the following spring [21,28]. Because feral horses maintain these basic social structures, they are a suitable model for studying whether social grooming contributes to the strengthening of affiliations within the herd.

Grooming behavior is broadly divided into self-grooming (auto-grooming) and mutual grooming (allo-grooming) [29,30,31]. Self-grooming consists of tail swishing, rolling, nipping, and rubbing on inanimate objects [29,32]. Mutual grooming generally occurs between two horses. They stand parallel to each other on the same side of the body and bite, lick, or pull their opponent’s head, mane, back, and hindquarters [16,23,33]. Mutual grooming enables each participant to clean body parts they cannot reach on their own and lowers participant heart rates [14]. In horses, mutual grooming may be a typical affiliative behavior [3,34] that strengthens long-term social relationships between unrelated mares [35] and is conciliatory after aggressive interactions [36]. There are multiple different hypotheses about the function of mutual grooming in horses. Adult feral mares may form affiliative relationships with other unrelated mares and stallions by mutual grooming, thereby reducing the number of aggressive interactions [2,26]. In Camargue horses in France, mutual grooming has never been observed between adult stallions but occurs between a stallion and a mare during the breeding season [14]. It is suggested that affiliative relationships between unrelated mares or stallions in a herd may increase reproductive success by reducing harassment from other mares or stallions [37]. Thus, it is hypothesized that strengthening the affiliation between unrelated horses by mutual grooming may increases reproductive success [2,35,38]. However, few empirical investigations have determined whether individuals that mutually groom have fewer aggressive interactions and build stronger affiliative relationships than individuals that do not mutually groom [3].

Affiliative relationships between pairs of horses may be indicated by the frequency of their close interactions [16,33,35]. Kinship and mutual grooming between herd members strengthens affiliative relationships [2,16,24]. It has been hypothesized that mutual grooming strengthens affiliative relationships between participating individuals [3,35]. Thus, a positive correlation is expected between mutual grooming frequency and proximity within the herd. Nevertheless, these parameters may differ between related and unrelated horses [2,39]. Therefore, it is necessary to control for pair kinship to verify this correlation [40].

Another hypothesis proposes that mutual grooming restores worsened relationships when aggressive interactions occur in pairs [36]. Thus, a positive correlation is expected between the frequency of aggressive interactions and mutual grooming in each pair. However, the frequency of aggressive interactions may be negatively correlated with the degree of kinship in each pair. Thus, it is necessary to control for pair kinship to verify this correlation.

Aggressive interactions may be byproducts of the physical proximity between individuals of different social rank [41,42]. In this case, a positive correlation is expected between the frequency of aggressive interactions and pair proximity. However, it is necessary to control for kinship to validate this prediction.

Grooming is considered a maintenance behavior in horses. It removes ectoparasites such as lice and flies [32]. The grooming parasite removal hypothesis [29,32] suggests that mutual grooming removes parasites that cannot be removed by self-grooming alone. A negative correlation is expected between the total grooming time that each individual receives from other individuals (mutual grooming) and the total time spent for self-grooming.

The objective of the study was to clarify the role of mutual grooming within the feral herd by testing the aforementioned predictions derived from three hypotheses regarding the function of mutual grooming; the affiliative relationship strengthening hypothesis, the worsened relationship restoring hypothesis, and the grooming parasite removal hypothesis.

## 2. Materials and Methods

### 2.1. Study Sites, Target Herd, and Study Period

Misaki feral horses live in a protected ~550 ha area at Cape Toi, Kushima City, Miyazaki Prefecture, Japan [21,22,43]. The ranch there was opened in 1697 during the Edo era. The horses have bred in a half-wild environment with minimal human modification or intervention. Thus, original social constructs in behavior of feral horses can be observed there. The frequency of aggressive interactions between individual Misaki feral horses is low as they have sufficient food resources and living space throughout all seasons except winter [21,44]. Moreover, stallions form new herds by sequestering mares not belonging to any particular group [22]. Herd size, structure, stability [21,45,46], and social rank among Misaki feral horses [21] have been reported. Membership in each herd is stable throughout all seasons except winter [21,28,46]. However, no studies have considered the affiliative relationships and interactions within the herd. Many of the horses are accustomed to human observers and have been individually identified [21]. For these reasons, Misaki feral horses are considered to be suitable subjects for the analysis of individual herd member behavior and interactions between pairs in specific seasons.

As of 2018, there are 120 extant Misaki feral horses. They inhabit Komatsugaoka and Ogiyama, located at altitudes of 287 m and 296 m, respectively, situated near the center of Cape Toi, Kushima City, Miyazaki Prefecture, Japan. The aforementioned regions have the largest total grassland area within the cape and constitute the main home range of Misaki feral horses [21]. The Misaki feral horse population is classified into 20 herds, several bachelor herds, and solitary bachelors. Individual identification, herd membership and composition, births, deaths, and maternal relationships have been recorded and made available by the research team of Miyazaki University for several decades.

One of the herds (the “6m” group) uses Komatsugaoka as their home range. All the nine members of the “6m” group were targeted for this survey (Table S1). During the study period, the “6m” group consisted of two stallions (“6m” and “16m”), one young adult male (“63m”), four unrelated adult mares (“73f,” “90f,” “20f,” and “94f”), one colt (juvenile male) (“76m”), and one filly (juvenile female) (“75f”). No other members joined or left the herd during the survey.

The study was conducted during breeding season and the beginning of the non-breeding season of the Misaki feral horse [21,43]. The observation was planned to be conducted between 9:00 a.m. and 4:00 p.m. for 1–4 h per day, depending on the weather conditions. As a result, the observation was conducted for seven days for a total of 16.5 h on 9–17 May 2018, three days for a total of 6.0 h on 1–4 July 2018, and five days for a total of 11.0 h on October 4–8, 2018. The total observation time of the “6m” group was 33.5 h and the mean daily observation time (± SD) was 2.09 ± 0.85 h (15 survey days).

### 2.2. Behavioral Data Collection and Treatment

#### 2.2.1. Kinship

The kinship of the paternal lineage of Misaki feral horses is unknown. However, the maternal lineage was elucidated through birth records. The relatedness (r) among the members of the “6m” group was assumed to be 0.5 for individuals in mother-child relationships, and 0.25 for individuals in sibling relationships sharing a mother. The relatedness between individuals without a directly confirmed kinship was assigned to 0. A kinship matrix was constructed wherein the relatedness between pairs was used as each element.

The Misaki feral horses were highly aggregated during the study period [21,43,46]. N.S., the second author, tracked the “6m” group with a digital Handycam (SONY HDR-CX680; Sony Corporation, Tokyo, Japan) to videotape all members in the field of view simultaneously. Except during inclement weather, the herd was followed for ~2 h each morning and afternoon daily. All self- and mutual grooming and aggressive interactions occurring within the herd were continuously recorded by the all occurrence sampling method [47].

#### 2.2.2. Grooming

For both self- and mutual grooming, the start and end times and the individual who started and ended the behavior were recorded. The total time spent self-grooming by each individual was calculated in seconds from these data. Mutual grooming is expressed in terms of the asymmetrical state wherein individual *i* grooms individual *j* but not vice-versa, the symmetrical state wherein both *i* and *j* are grooming each other simultaneously, and the pausing state defined as an interruption in grooming that is resumed within 120 s. If mutual grooming was resumed >120 s after the interruption, it was defined as an independent bout. For each individual, the total amount of grooming received from other individuals during mutual grooming was reported as the sum of the asymmetrical and symmetrical states. A grooming matrix was constructed using the number of observed mutual grooming bouts throughout the study period in all pairs as elements.

#### 2.2.3. Aggression

In aggressive interactions, an individual threatens or attacks another individual and/or the threatened or attacked individual retreats, flees, or avoids [23,30,36]. Threats include arched necks, stand-stares, and ears-laid-back. Attacks include biting, balking, and chase kicking [23,48]. For each aggressive interaction, when the individual being threatened or attacked escaped or avoided the attack, it was recorded as the loser and the attacking side was recorded as the winner. The interaction was scored a draw if an individual did not respond to an attack by another horse or escaped or avoided approaching the individual in the absence of an attack or threat. A winner/loser matrix was constructed by plotting the number of aggressive interaction wins, losses, and draws between pairs. An aggression matrix was plotted by using the number of aggressive interactions between pairs as elements.

The relative social rank among the members of the “6m” group was scaled from a winner/loser matrix by the Batchelder–Bershad–Simpson (BBS) method [49]. The s(*i*) of individual *i* was calculated according to Equation (1) and each s(*i*) was corrected according to Equation (2) to obtain s′(*i*). Individuals with a larger s’(*i*) were regarded as higher-ranked:

(1) $$s\left(i\right)=\frac{\sqrt{2\pi }\left(2W_{i}-N_{i}\right)}{2N_{i}}$$

(2) $$s^{\prime }\left(i\right)=\left[\frac{2\left(W_{i}-L_{i}\right)}{N_{i}}\right]+Q_{i}$$

where *W_i_* is the total number of aggressive interactions won by individual *i*, *N_i_* is the total number of aggressive interactions involving *i*, *L_i_* is the total number of aggressive interactions lost by *i*, and *Q_i_* is the average *s*(*i*) for all individuals engaged in aggressive interactions with *i*.

#### 2.2.4. Proximity Matrices, Social Rank, and Social Network Analysis

The nearest neighbor of each individual was recorded at 5 min intervals by the instantaneous sampling method [46]. The proximity index (*PI_ij_*) of a pair of individuals *i* and *j* was defined as a simple proximity index [40] and calculated using Equation (3). The *PI* of each pair has a value from 0–1 and indicated the relative height of the proximity frequency for the pair in the “6m” group [40]. It represents the relative strength of the affiliative relationship of the pair [16,33,35].

(3) $$PI_{ij}=\frac{C_{i}\left(j\right)+C_{j}\left(i\right)}{T_{i}+T_{j}}$$

where *C_i_*(*j*) is the total number of sample points where *j* is the nearest neighbor to *i*, and *T_i_* is the total number of sample points for *i*. A proximity matrix was constructed using the *PI* among all pairs as elements.

The social network was based on the proximity relationship between pairs in the “6m” group. In a herd comprising nine individuals, there were 36 (= 9 × 8/2) pairs or combinations of two members. The *PI*s were filtered such that only the values exceeding the mean + SD of all *PI*s for 36 pairs remained. These pairs were regarded as the proximity relationships representing the “6m” group [40]. A social network was constructed according to the filtered *PI*s. The eigenvector centrality for each individual was calculated according to the social network.

### 2.3. Statistical Analyses

The relationship between mutual grooming and proximity for each pair was evaluated. The correlation between the grooming and proximity matrices was determined using Kendall’s tau correlation test (Tau *Kr* test). The kinship matrix served as the control and the correlation between the aforementioned matrices was then calculated with a matrix rank partial correlation test (partial Tau *Kr* test) [50,51]. The relationships between aggressive interactions and pair proximity, and between mutual grooming and aggressive interactions were examined as described above. For each test, the row or column randomization number was 100,000.

The relationship between the amount of self-grooming and mutual grooming of each individual, as well as the relationship between centrality in the social network and social rank of each individual, was examined by regression analysis (*F*-test). In the former analysis, the objective variable was the total time of self-grooming observed for each individual and the explanatory variable was the total time of grooming received from other individuals during mutual grooming. In the latter analysis, the objective variable was the eigenvector centrality of each individual and the explanatory variable was *s*’.

All data were processed using the statistical freeware HAD [52], UCINET, NetDraw 2.166 [53], and MatrixtesterPrj [50]. The significance level was 0.05.

### 2.4. Ethical Approval

Our research was approved by the Teikyo University of Science Animal Committee (No. 18C015) and permitted by Kushima City, Miyazaki Prefecture. Our behavioral research adhered to the guidelines of the International Society for Applied Ethology (http://www.applied-ethology.org/ethicalguidelines.htm) for the ethical use of animals.

## 3. Results

### 3.1. Mutual Grooming Interaction Structure

The kinship, mutual grooming, aggression, and proximity matrices are shown in Table 1, Table 2, Table 3 and Table 4, where rows and columns refer to consecutive names given to males (m) and females (f).

Self-grooming was observed in all “6m” herd members. The mean total time (± *SD*) spent by each individual in self-grooming during the study period was 2174.8 ± 1346.6 s (*N* = 9). (Table S2).

Mutual grooming was confirmed for 6/9 horses: two stallions (“6m” and “16m”), three adult mares (“20f,” “73f,” and “90f”), and one colt (“76m”). There were 84 bouts (Table 2). All observed mutual grooming bouts occurred only between two individuals from start to finish. In no instance did a third individual join a grooming pair. The mean total time (± *SD*) spent by each individual in self-grooming during the study period was 477.0 ± 351.5 s (*N* = 9) (Table S2). The mean duration of a single mutual grooming bout was 84.8 ± 80.5 s (*N* = 34; range 3–314 s). In one bout, the symmetrical state (62.4 ± 54.7 s) was linked to a pausing state (21.0 ± 33.7 s) or short asymmetrical state (1.4 ± 4.0 s). The total duration of symmetrical and asymmetrical grooming was 2123 s (97.8%) and 47 s (2.2%), respectively.

There was a significant negative correlation between the total time each individual spent in self-grooming and the total time each individual was groomed by another in mutual grooming (Table S2: *β* = −0.195; *R*^2^ = 0.559; *F*_1,7_ = 8.888; *p* = 0.020).

### 3.2. Social Relationships and Interactions Among “6m” Herd Members

A winner/loser matrix is shown in Table S3. The *s’* for each member was calculated from the winner/loser matrix by the BBS method. Both *s’* and the social rank based on *s’* are shown in Table S2. “6m” was the first-ranked individual. The *s’* values of the second- to fourth-ranked individuals did not differ and were classified into the upper-ranked group (“73f,” “90f,” and “16m”). The fifth- and sixth-ranked individuals were categorized into the middle-ranked group (“20f” and “94f”). The seventh- to ninth-ranked individuals were placed in the lower-ranked group (“63m,” “76m,” and “75f”) (Table S2).

**Figure 1:**
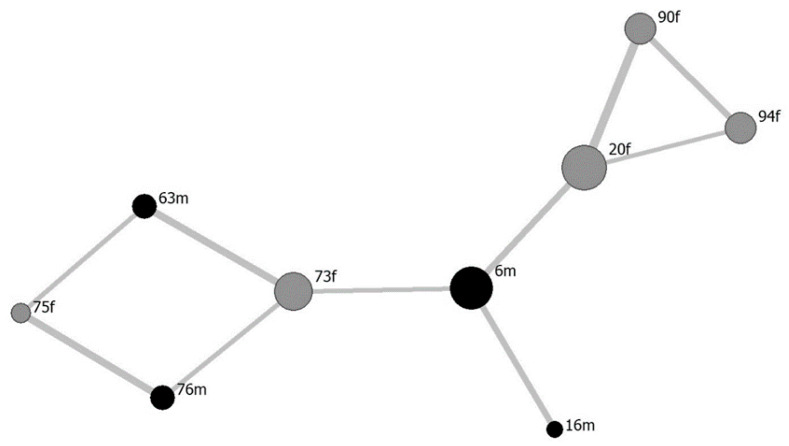
Social network based on proximity index of the “6m” herd. Black points represent males; “6m” and “16m” are stallions, “63m” is young male, and “76m” is a colt. Gray points represent mares; “73f,” “90f,” “20f,” and “94f” are mares, and “75f” is a filly. Size of the point represents the magnitude of the centrality of the eigenvector and thickness of the line represents the degree of proximity between individuals.

The *PI*s among all “6m” members were >0 (Table 3). For ten pairs, *PI* > mean + SD (= 0.1387). According to the *PI* between pairs, the social network of the “6m” group was structured such that the first-ranked male (“6m”) occupied the central position and connected to three different subgroups (Figure 1). The first subgroup consisted of “16m,” which was the second-ranked male. It was observed that “6m” repeatedly and frequently directed aggressive behavior against “16m” and ultimately drove him out of the herd. The second subgroup comprised the related herd members, namely, a mother (“73f”) and her offspring (“63m,” “75f,” and “76m”). The third subgroup contained three unrelated mares (“20f,” “90f,” and “94f”). The *PI* between “20f” and “90f” was the highest for the entire “6m” group (Table 3). No significant correlation was found between the social network eigenvector centrality and the rank (*s’*) of each individual (*β* = 0.063; *R*^2^ = 0.330; *F*_1,7_ = 3.448; *p* = 0.106).

### 3.3. Tau Kr Test

**Figure 2:**
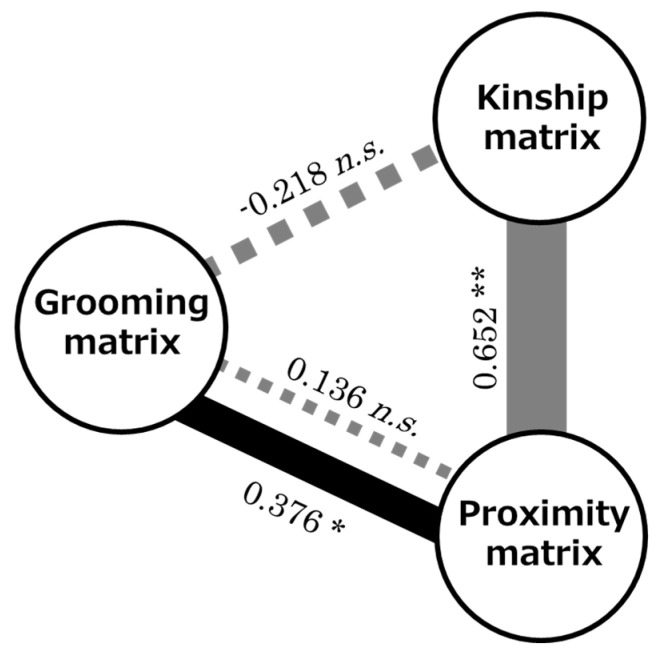
Correlation diagram of the matrices. Solid gray lines and the boldness indicate significant matrix rank correlations and coefficients (Tau *Kr*), respectively, dotted lines indicate non-significant correlations, and black lines and the boldness indicate partial correlations and coefficients, respectively. The respective coefficients are shown above the line. * indicates *p* < 0.05 and ** indicates *p* < 0.01. *n. s.* denotes a nonsignificant (*p* > 0.05).

No significant correlation was found between the kinship and grooming matrices (Figure 2: Tau *Kr* = −0.218; *p* = 0.3040). A significant negative correlation was found between the kinship and aggression matrices (Tau *Kr* = −0.382; *p* = 0.0498). A significant positive correlation was found between the kinship and proximity matrices (Figure 2: Tau *Kr* = 0.652; *p* = 0.0079).

No significant correlation was found between the grooming and proximity matrices (Figure 2: Tau *Kr* = 0.136; *p* = 0.2050); however, a positive partial correlation was found between them when correcting for kinship (Figure 2: partial Tau *Kr* = 0.376; *p* = 0.0154). The correlation between the aggression and grooming matrices was not significant (Tau *Kr* = −0.061; *p* = 0.3687). No partial correlation was found between them even after kinship correction (partial Tau *Kr* = −0.160; *p* = 0.2049). No significant positive correlation was found between the aggression and proximity matrices (Tau *Kr* = 0.047; *p* = 0.3364); however, a significant partial correlation was found between them after correcting for kinship (partial Tau *Kr* = 0.422; *p* = 0.0003).

## 4. Discussion

### 4.1. Social Structure and Social Ranking in the “6m” Herd

The social network of the “6m” group based on its proximity relationships had a structure centered on “6m,” who was connected to three different subgroups. Individuals with higher ranks did not necessarily have higher group eigenvector centrality. Only those in the upper ranks were in proximity to “6m/” The middle-ranked individuals, “90f” and “94f,” maintained proximity to “6m”, mediated by the upper-ranked individual “20f.” All three lower-ranked individuals were siblings of ‘73f’ and had proximity to each other. The proximity between “73f” and “6m” indirectly maintained those between the lower-ranked individuals and “6m” (Figure 1). The closest proximity was found between the oldest female “20f” and the unrelated young adult female “90f.” Female mutual grooming was confirmed only between “20f” and “90f.”

These results corroborate those of previous reports. Individual feral horses of similar rank on Yururi Island in Hokkaido maintain proximity to each other [16,24,33], as do Misaki feral horses [21]. Horses forming strong affiliative relationships might mutually groom each other within a herd [16]. These results suggest that during the study period, the group with “6m” as the highest rank individual was a typical herd with characteristics resembling those described in previous studies.

### 4.2. Mutual Grooming And Affiliative Relationships between Horses

A significant positive partial correlation was found between the grooming and proximity matrices when kinship was controlled. This finding upholds the affiliative relationship strengthening hypothesis. A significant positive correlation was also found between the aggression and proximity matrices when kinship was controlled. This result supports the hypothesis that proximity facilitates aggressive interactions between pairs. In contrast, no significant correlation was found between the aggression and grooming matrices here and the worsened relationship restoring hypothesis was not corroborated.

On Yururi Island in Japan, the individual free-ranging horses most often in proximity differ from those frequently engaging in mutual grooming [16]. The current results were similar to those of the aforementioned study but only when not correcting for kinship. The proximity and grooming matrices were not correlated. However, the horses on Yururi Island were members of a family herd [30]. Therefore, it is presumed that they were all related to each other and it would be impossible to analyze their proximity and mutual grooming while controlling for their kinship. When correcting for kinship in the present study, a positive correlation between proximity and mutual grooming was found. This discovery corroborates a previous report stating that proximity is correlated with mutual grooming between pairs of horses in the Serra d’Arga Mountain of Portugal [54].

If mutual grooming frequency increases with pair proximity, then mutual grooming frequency and aggressive interactions should be positively correlated. However, no significant correlation was found between aggression and mutual grooming between members of the “6m” group in the present study. The results of this study did not enable the estimation of the causal relationships of the effects of interactions between pairs on affiliative relationships. Pairs of related or unrelated individuals that frequently participate in mutual grooming have closer proximity [16]. Consequently, the risk of aggressive interactions increases between individuals differing in rank and physical proximity [42]. The first-ranked male “6m” and the second-ranked male “16m” were in proximity, but the former unilaterally attacked and repelled the latter, and there was no mutual grooming between them. Proximity, then, implies the potential for aggressive interactions between members of a pair and depends on the pair combination rather than their affiliative relationship.

The present study also upheld the parasite removal hypothesis. Individuals that spent less time on self-grooming invested longer times receiving grooming from other individuals. This discovery may suggest that mutual grooming has not only an aforementioned social function, such as strengthening affiliative relationships, but also a maintenance function, such as parasite removal [31,32].

In mutual grooming, both horses benefit from hygienic maintenance as they both groom and are groomed [55,56]. Here, mutual grooming between members of the “6m” group was performed symmetrically for the majority of the observations. Thus, mutual grooming between horses was almost completely and perfectly symmetrical. One horse received grooming from the other as soon as the former started grooming the latter. As soon as one horse stopped grooming, the other stopped as well. These behaviors applied both to related and unrelated pairs of horses. In all cases, the horses faced each other and mutually groomed nearly the same body parts simultaneously [16,33]. In this manner, each animal would receive an equal benefit regarding the simultaneous removal of parasites [56]. However, since this study did not take into account the different parasite loads for different individuals, receiving the same amount of mutual grooming may not be of the same benefit to all individuals. Future research needs to determine the amount of mutual grooming that takes into account the parasite load of each individual.

Mutual grooming between two primates strengthens affiliative relationships as they must both actively maintain interaction symmetry [1,4,5,56]. Thus, reciprocal altruism is characteristic of primate grooming. In contrast, asymmetrical social grooming is relatively less effective at strengthening affiliative relationships in species forming groups in which memberships rapidly and/or frequently fluctuate. The members of Misaki feral horse herds may change annually and numerous mares have indeterminate herd affiliations [16,43,46]. Symmetrical mutual grooming might ensure immediate parasite removal reciprocity and help forge affiliative relationships between unrelated individuals.

## 5. Conclusions

The present study supported the strengthening affiliative relationship and parasite removal hypotheses but did not corroborate the relationship restoring hypothesis. However, this study is merely a single case report based on relatively short observations made for one Misaki feral horse herd group when they were highly aggregated during a particular season. The aforementioned hypotheses must be tested on multiple herds and in longer-term observations of Misaki and other feral horses worldwide and can be extrapolated to understand behavioral stereotypies in horses in intensive production systems. The findings of this study suggest that comparisons between horses and other taxa, such as primates, may effectively elucidate the mechanisms by which interactions between group members maintain and strengthen their social relationships [15,54,57].

## Figures and Tables

**Table 1:** Kinship matrix.

	6m	16m	63m	20f	73f	90f	94f	75f	76m
6m		0	0	0	0	0	0	0	0
16m			0	0	0	0	0	0	0
63m				0	0.5	0	0	0.25	0.25
20f					0	0	0	0	0
73f						0	0	0.5	0.5
90f							0	0	0
94f								0	0
75f									0.25
76m									

**Table 2:** Grooming matrix (mean = 0.944; SD = 0.472).

	6m	16m	63m	20f	73f	90f	94f	75f	76m
6m		0	0	6	4	1	0	0	1
16m			0	0	3	3	0	0	3
63m				0	0	0	0	0	0
20f					0	6	0	0	1
73f						0	0	0	2
90f							0	0	4
94f								0	0
75f									0
76m									

**Table 3:** Proximity matrix (mean = 0.125; SD = 0.014).

	6m	16m	63m	20f	73f	90f	94f	75f	76m
6m		0.2383	0.0622	0.2313	0.1775	0.1225	0.1328	0.0687	0.0523
16m			0.0359	0.0514	0.0982	0.0741	0.1166	0.0734	0.0619
63m				0.1274	0.2988	0.0691	0.1294	0.1843	0.1357
20f					0.0544	0.3393	0.1601	0.0839	0.0660
73f						0.0798	0.0779	0.1141	0.1769
90f							0.2323	0.0705	0.0566
94f								0.0967	0.0599
75f									0.2889
76m									

**Table 4:** Aggression matrix (mean = 4.444; SD = 0.722).

	6m	16m	63m	20f	73f	90f	94f	75f	76m
6m		24	10	13	9	7	14	6	0
16m			4	3	1	3	3	2	4
63m				0	0	5	10	1	2
20f					2	6	0	0	0
73f						6	3	1	0
90f							12	4	2
94f								1	1
75f									1
76m									

## Supplementary Materials

The following are available online at https://www.mdpi.com/2076-2615/10/9/1564/s1, Table S1. Sex, age at time of study period, kinship, and total observation time for “6m” herd members.; Table S2. Frequency of aggressive interaction, s’, eigenvector centrality, and total durations of self-grooming (min) and mutual grooming (min); Table S3. Winner/loser matrix. Left column shows name of winning individual. Top row shows name of losing individual. Numerical value at upper right of diagonal component indicates number of wins. Numerical value at lower left indicates number of losses—indicates no aggressive interaction observed in pair. The number in parentheses represents the number of aggressive interactions that ended in a draw.
