# Supplementary material for: The Contribution of Mutual Grooming to Affiliative Relationships in a Feral Misaki Horse Herd
Source: Animals (Basel). 2020 Sep 3;10(9):1564. doi: 10.3390/ani10091564 (PMC7552250; doi:10.3390/ani10091564)
Supplement: Supplementary file 1 [file animals-10-01564-s001.pdf]

animals

Supplementary Data

Supplemental Table S1. Sex, age at time of study period, kinship, and total observation time for ‘6m’ herd members.

Name	Sex	Age	Kinship	Total observation hours
6m	M	12		33.45
73f	F	8	a mother of 63m, 76m, 75f	33.18
90f	F	5		32.16
16m	M	11		32.44
20f	F	16		32.85
94f	F	4		33.40
63m	M	5	a son of 73f	32.78
76m	M	1	a son of 73f	32.49
75f	F	2	a daughter of 73f	32.55

Supplemental Table S2. Frequency of aggressive interaction, *s*′, eigenvector centrality, and total durations of self-grooming (sec) and mutual grooming (sec).

Name	frequency of aggressive interaction (times/h)	*s*′	Eigenvector centrality	Total time spent for self grooming (sec)	Total time spent for mutual grooming (sec)
6m	1.73	0.93	0.438	1060	512
73f	0.65	-0.46	0.399	1246	668
90f	1.13	-0.57	0.333	3050	637
16m	1.18	-0.58	0.183	1100	794
20f	0.59	-1.02	0.463	607	829
94f	1.29	-1.20	0.333	4742	0
63m	0.85	-1.72	0.257	3914	0
76m	0.28	-1.83	0.257	1707	853
75f	0.49	-1.95	0.216	2147	0

Supplemental Table S3. Winner/loser matrix. Left column shows name of winning individual. Top row shows name of losing individual. Numerical value at upper right of diagonal component indicates number of wins. Numerical value at lower left indicates number of losses. - indicates no aggressive interaction observed in pair. The number in parentheses represents the number of aggressive interactions that ended in a draw.

		**loser**								
	**Name**	**6m**	**73f**	**90f**	**16m**	**20f**	**94f**	**63m**	**76m**	**75f**
**winner**	**6m**		**8(0)**	**6(1)**	**16(8)**	**12(1)**	**13(1)**	**8(2)**	**-**	**5(1)**
	**73f**	**1(0)**		**3(2)**	**0(0)**	**2(0)**	**3(0)**	**-**	**-**	**1(0)**
	**90f**	**0(1)**	**1(2)**		**0(0)**	**0(3)**	**7(5)**	**3(2)**	**1(1)**	**3(1)**
	**16m**	**0(8)**	**1(0)**	**3(0)**		**1(2)**	**2(1)**	**3(1)**	**4(0)**	**2(0)**
	**20f**	**0(1)**	**0(0)**	**3(3)**	**0(2)**		**-**	**-**	**-**	**-**
	**94f**	**0(1)**	**0(0)**	**0(5)**	**0(1)**	**-**		**8(2)**	**0(1)**	**1(0)**
	**63m**	**0(2)**	**-**	**0(2)**	**0(1)**	**-**	**0(2)**		**1(1)**	**1(0)**
	**76m**	**-**	**-**	**0(1)**	**0(0)**	**-**	**0(1)**	**0(1)**		**1(0)**
	**75f**	**0(1)**	**0(0)**	**0(1)**	**0(0)**	**-**	**0(0)**	**0(0)**	**1(0)**	
